# WNT/*β*-Catenin Signaling Is Required for Integration of CD_24+_ Renal Progenitor Cells into Glycerol-Damaged Adult Renal Tubules

**DOI:** 10.1155/2015/391043

**Published:** 2015-05-18

**Authors:** Zhao Zhang, Diana M. Iglesias, Rachel Corsini, LeeLee Chu, Paul Goodyer

**Affiliations:** ^1^Department of Paediatrics, Montreal Children's Hospital Research Institute, McGill University, Montreal, QC, Canada; ^2^Department of Human Genetics, Montreal Children's Hospital Research Institute, McGill University, Montreal, QC, Canada

## Abstract

During development, nephron progenitor cells (NPC) are induced to differentiate by WNT9b signals from the ureteric bud. Although nephrogenesis ends in the perinatal period, acute kidney injury (AKI) elicits repopulation of damaged nephrons. Interestingly, embryonic NPC infused into adult mice with AKI are incorporated into regenerating tubules. Since WNT/*β*-catenin signaling is crucial for primary nephrogenesis, we reasoned that it might also be needed for the endogenous repair mechanism and for integration of exogenous NPC. When we examined glycerol-induced AKI in adult mice bearing a *β*-catenin/TCF reporter transgene, endogenous tubular cells reexpressed the NPC marker, CD24, and showed widespread *β*-catenin/TCF signaling. We isolated CD_24+_ cells from E15 kidneys of mice with the canonical WNT signaling reporter. 40% of cells responded to WNT3a *in vitro* and when infused into glycerol-injured adult, the cells exhibited *β*-catenin/TCF reporter activity when integrated into damaged tubules. When embryonic CD_24+_ cells were treated with a *β*-catenin/TCF pathway inhibitor (IWR-1) prior to infusion into glycerol-injured mice, tubular integration of cells was sharply reduced. Thus, the endogenous canonical *β*-catenin/TCF pathway is reactivated during recovery from AKI and is required for integration of exogenous embryonic renal progenitor cells into damaged tubules. These events appear to recapitulate the WNT-dependent inductive process which drives primary nephrogenesis.

## 1. Introduction

During early mammalian embryogenesis, uncommitted mesenchymal stem cells that will form the metanephric kidney downregulate genes marking pluripotentiality (e.g.,* Oct4*,* Nanog*) and begin to express a transcription factor, OSR1, that specifies the pool of renal progenitor cells (RPC) in intermediate mesoderm [1]. Fate-mapping studies in embryonic mice suggest that, by E9, some RPC give rise to the nephric ducts and their derivative ureteric buds [1]; others, expressing WT1, are committed to a nephron progenitor cell (NPC) phenotype and are primed for responsiveness to the inductive WNT signal. After embryonic day E11.5, WNT9b signals from the arborizing ureteric bud (UB) begin to induce NPC to cluster at the tip of each UB branch tip, exhibit robust *β*-catenin/TCF pathway signaling activity [2, 3], and then differentiate into epithelial cells of the emerging nephrons. WNT9b activation of the canonical WNT signaling pathway is essential for induction of RPC; ectopic WNT9b can substitute experimentally for the UB signal [3]. At the S-shaped body stage, canonical *β*-catenin/TCF signaling activity is sustained by autocrine expression of WNT4 in the differentiating cells [3], but pathway activity is downregulated as nephrons undergo terminal differentiation and renal development comes to an end [2].

Sagrinati et al. used the stem cell surface marker CD24 to track the fate of RPC in adult human and mouse kidney [4]. They found CD_24+_ cells both in the nephrogenic mesenchyme and in the ureteric bud, paralleling fate-mapping studies by Mugford which showed that a common early progenitor pool of* Osr1* (+) cells gives rise to both lineages [1]. In embryonic human kidney, CD_24+_ cells acquire CD133 at the cell surface when committed to the NPC phenotype. CD24/CD133 expression is sustained in nephrogenic cells of the early renal vesicle and S-shaped body [5]. The two surface antigens mark 35–50% of all cells in the human fetal kidney at 8-9-week gestation but only 10–20% of kidney cells by 12–17 weeks [6]. Interestingly, Lazzeri et al. isolated CD_24+_/CD_133+_ cells from human embryonic kidney and infused them into SCID mice at the peak of glycerol-induced acute kidney injury; this improved renal function and improved renal histology compared to controls (Lazzeri). Remarkably, after 2 weeks, labeled exogenous NPC constituted about 15% of proximal tubular cells and were continuing to proliferate within the tubular wall [6].

The mechanism by which exogenous embryonic RPC are integrated into the damaged adult renal tubule is unknown. However, we reasoned that the process might recapitulate events during kidney development and might also be related to the endogenous tubular repair mechanism in adult kidney. If so, regenerative events must recruit the canonical WNT/*β*-catenin/TCF signaling pathway that is crucial for primary nephrogenesis. To test this hypothesis, we induced glycerol-mediated proximal tubular injury in adult mice and then examined *β*-catenin/TCF signaling in endogenous cells and in infused exogenous embryonic nephron progenitor cells during their integration into damaged tubules.

## 2. Results

### 2.1. A Subset of Endogenous Renal Cells Reactivate Canonical *β*-Catenin/TCF Signaling after Glycerol-Induced Tubular Injury

**Figure 1:**
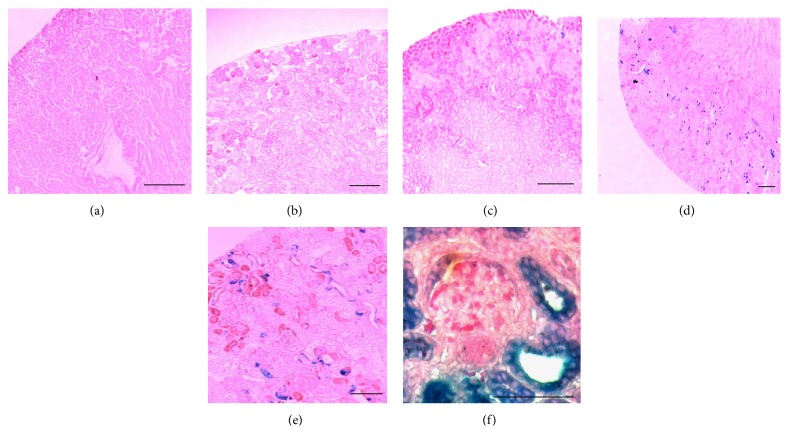
Endogenous *β*-catenin/TCF reporter transgene activity following glycerol-induced renal tubular injury in adult mice. (a and b) No *β*-galactosidase signal is seen in uninjured or glycerol-treated mouse kidneys of CD1 wild-type mice. (c) *β*-galactosidase signaling is minimal in the uninjured *β*-catenin/TCF reporter mouse kidney. (d–f) Strong *β*-catenin/TCF reporter activity (X-Gal, blue) is seen in renal tubules of adult mouse kidney 3 days after glycerol-induced renal injury. Scale bars: 50 *μ*m.

**Figure 2:**
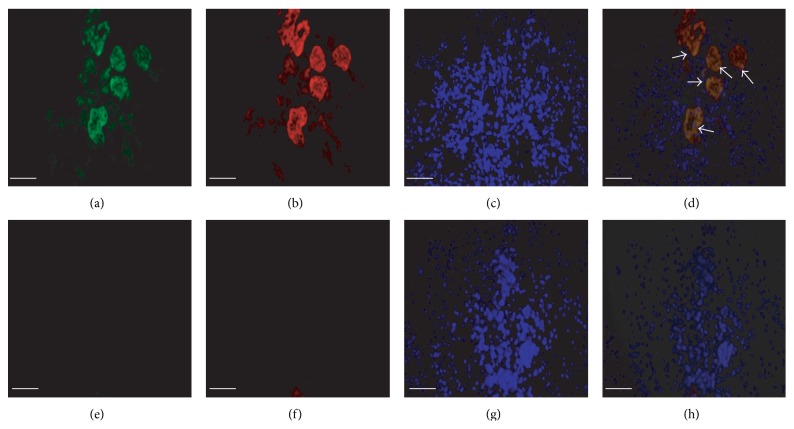
Endogenous coexpression of CD24 and *β*-catenin/TCF reporter transgene following glycerol-induced renal injury in adult mice. (a–d) Kidney sections showing endogenous tubular cells of glycerol-treated mice: (a) immunostaining for reporter *β*-galactosidase (green); (b) immunostaining for CD24 (red); (c) nuclear staining with DAPI; (d) merged images showing coexpression of *β*-galactosidase and CD24 in tubular cells (white arrows). (e–h) Kidney sections showing endogenous tubular cells in control (PBS injected) mice: (e) *β*-galactosidase reporter (green); (f) CD24 (red); (g) DAPI; (h) merge. Scale bars: 50 *μ*m.

During recovery from glycerol-induced injury, damaged proximal tubules are repopulated by proliferating endogenous cells. To ascertain whether this endogenous repair process engages the canonical WNT signaling pathway, we induced proximal tubular injury with 50% glycerol (8 *μ*L/g i.m.) in adult mice bearing a *β*-catenin/TCF-lacZ reporter transgene or wild-type CD1 mice [2]. No *β*-galactosidase signal was seen in uninjured (Figure 1(a)) or glycerol-treated (Figure 1(b)) wild-type mouse kidney or in uninjured *β*-catenin/TCF-lacZ reporter mice (Figure 1(c)) after three days. In contrast, we noted a strong reporter signal in tubular cells within glycerol-injured areas of the renal cortex among *β*-catenin/TCF-lacZ reporter mice (Figures 1(d)–1(f)). Most proximal tubular cells with a strong *β*-galactosidase signal also showed coexpression of the renal progenitor cell marker, CD24 (Figures 2(a)–2(d)).

### 2.2. CD_24+_ Renal Progenitor Cells from Embryonic Kidney Exhibit Canonical *β*-Catenin/TCF Signaling in Response to WNT3a* In Vitro*

**Figure 3:**
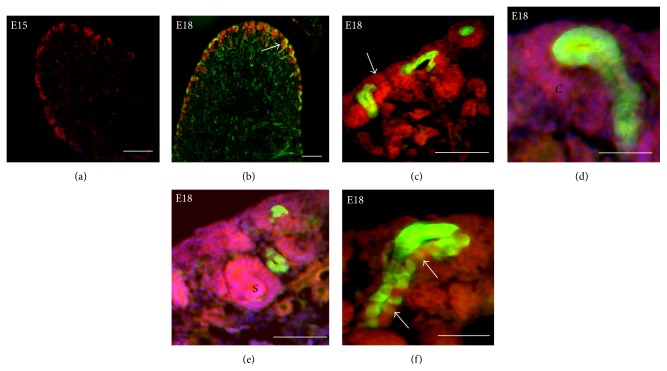
CD24 expression in embryonic mouse kidney. (a) PE-red-tagged CD_24+_ cells are seen predominantly in the nephrogenic zone of E15 mouse kidney. (b-c) In E18 kidney from* Hoxb7*-GFP transgenic mice, CD_24+_ cells (red) are seen in the cap mesenchyme associated with ureteric bud branch tips (green). (d-e) At high power, CD_24+_ cells (red) are seen within comma-shaped (C) and S-shaped (S) bodies of emerging nephrons. (f) Occasional CD_24+_ cells (red) are seen within the ureteric bud trunk (green) of E18* Hoxb7*-GFP mice. Scale bar: 50 *μ*m.

At embryonic day E15, the CD24 surface antigen is predominantly expressed in putative RPC of the nephrogenic zone (Figure 3(a)). To refine the distribution of these CD_24+_ cells, we examined E18 kidneys from mice bearing the* Hoxb7*-GFP transgene to mark the ureteric bud (Figures 3(b)–3(e)). CD_24+_ cells are seen in the cap mesenchyme surrounding ureteric bud tips (Figures 3(b) and 3(c)) and in the comma-shaped (Figure 3(d)) and S-shaped bodies (Figure 3(e)) of emerging nephrons. Some CD_24+_ cells are also seen scattered within the* Hoxb7*-GFP (+) ureteric bud trunk (Figure 3(f)).

**Figure 4:**
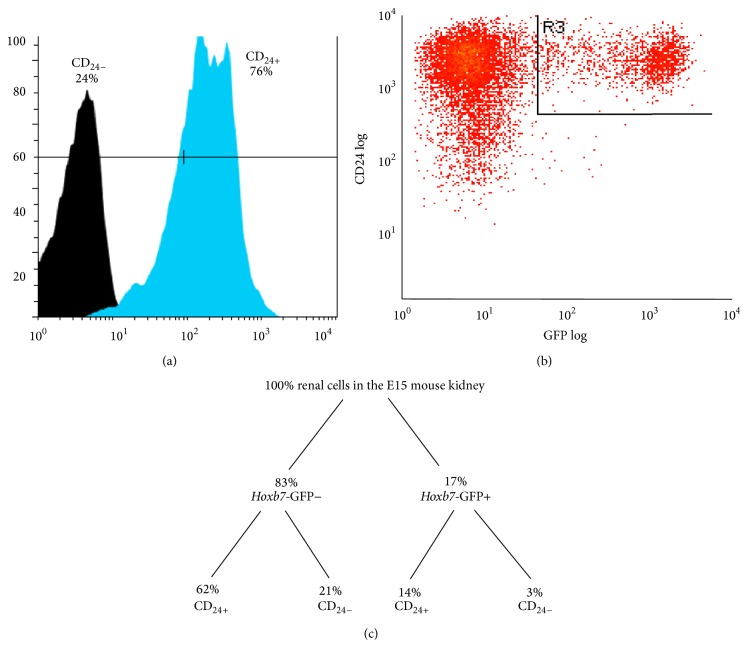
FACS isolation of CD_24+_ cells from E15 mouse kidney. Single-cell suspensions were prepared from E15* Hoxb7*-GFP mouse kidneys and analyzed for expression of CD24 and* Hoxb7*-GFP by fluorescence-activated cell sorting (FACS). (a) CD_24+_ cells represent about 76% of cells in the E15 mouse kidney. (b) 14% of CD_24+_ cells coexpress the* Hoxb7*-GFP ureteric bud marker. (c) Percentages of E15 mouse kidney cells expressing CD24/*Hoxb7*-GFP.

To characterize the responsiveness of CD_24+_ cells to canonical WNT signals, we excised kidneys from embryonic day E15* Hoxb7*-GFP mice and isolated CD_24+_ cells by fluorescence-activated cell sorting (FACS, blue peak) (Figure 4(a)). About 14% of the CD_24+_ cells also expressed GFP (Figures 4(b) and 4(c)). To characterize the CD_24+_ cells, we replated them in monolayer culture for 48 hours and examined expression of various developmental genes by RT-PCR. We identified transcripts for multiple markers of the metanephric mesenchyme (*Wt1*,* Osr1*, and* Gdnf*), cap mesenchyme (*Six2*,* Cited1*), and S-shaped body (*Wnt4, Pax8)*. We also identified expression of genes associated with the UB trunk (*Wnt7b*) but not the UB tip (e.g.,* Wnt9b*,* Ret*, and* Wnt11*).

**Figure 5:**
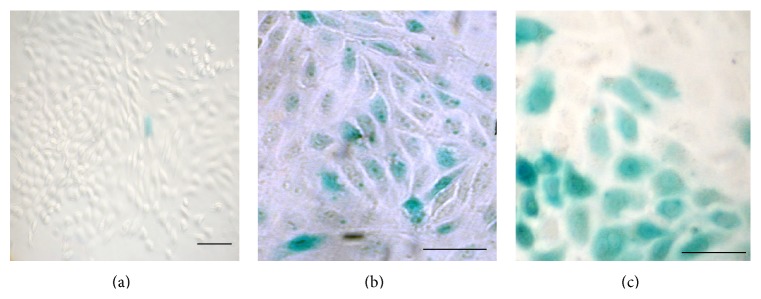
*β*-catenin/TCF signaling in CD_24+_ embryonic kidney cells* in vitro*. (a) CD_24+_ cells isolated from E15 kidney of mice bearing the *β*-catenin/TCF-lacZ transgene show minimal *β*-galactosidase activity (blue) in baseline monolayer culture. (b) About 40% of CD_24+_ cells exposed to murine L-cells expressing Wnt3a showed canonical *β*-catenin/TCF signaling. (c) CD_24+_ cells exposed to ureteric bud cells isolated by FACS from E15* Hoxb7*-GFP mice showed similar canonical *β*-catenin/TCF signaling. Scale bar: 50 *μ*m.

To examine activation of the canonical WNT signaling pathway, we isolated CD_24+_ cells by FACS from pooled E15 kidneys of progeny from mice bearing the *β*-catenin/TCF reporter transgene. After 24–48 hours in monolayer culture, few of the cells exhibited baseline reporter transgene activity (Figure 5(a)). However, when the CD_24+_ cell monolayer was cocultured with inserts containing L-cells expressing WNT3a or GFP(+) cells isolated from E15* Hoxb7*-GFP mice, about 40% of cells showed canonical *β*-catenin/TCF signaling activity (Figures 5(b) and 5(c)).

### 2.3. Infused CD_24+_ Cells from E15 Mouse Kidney Are Integrated into the Damaged Renal Tubules of Adult Mice with Glycerol-Induced Proximal Tubular Injury

**Figure 6:**
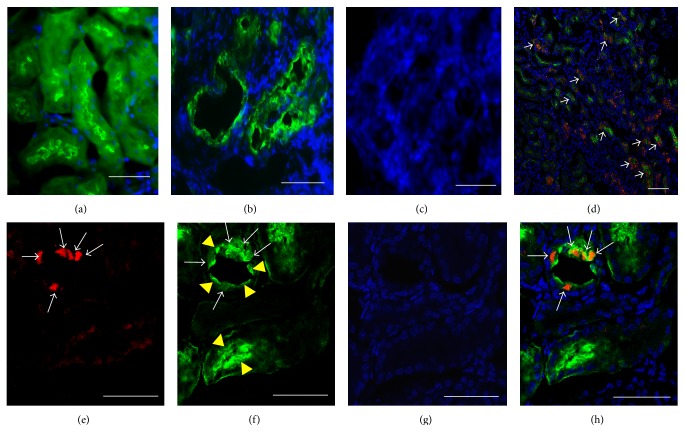
Exogenous renal embryonic CD_24+_ cells stained with PKH26 red integrate into the renal tubules of adult mice with glycerol-induced proximal tubular injury. (a)* Lotus tetragonolobus* agglutinin (LTA) staining of normal control renal proximal tubules (green). (b) LTA staining of kidney (green) 3 days after induction of acute tubular injury with 50% glycerol 8 *μ*L/g shows tubular dilatation, flattening, and detachment of proximal tubular cells. (c) No uptake of red dye is seen in glycerol-injured kidneys following infusion of the supernatant from the PKH26 red staining procedure for CD_24+_ cells. (d) Following infusion of PKH26 red-stained CD_24+_ cells into glycerol-injured mice, exogenous cells are seen within the damaged renal tubules (white arrows). (e–h) Confocal immunofluorescent microscopy examining integration of CD_24+_ cells into the wall of glycerol-damaged proximal tubules. (e) Patchy integration of PKH26 red CD_24+_ cells into the proximal tubular wall (white arrows). (f) Exogenous CD_24+_ cells (white arrows) have a polarized appearance and stain for LTA (green) and are intercalated among the endogenous LTA(+) epithelia (yellow arrow heads). (g) The same section stained with DAPI alone. (h) Merged image of (e), (f), (g). Scale bars: 50 *μ*m (f) and 70 *μ*m (g–j). LTA: green; DAPI: blue; PKH26: red.

To confirm that embryonic CD_24+_ cells can function as RPC and are integrated into acutely damaged renal tubules of adult mice, we first isolated CD_24+_ cells by FACS from kidneys of wild-type embryonic day E15 mice and stained them with PKH26 red fluorescent dye. We then induced proximal renal tubular injury with intramuscular injection of 50% glycerol (8 *μ*L/g body weight) in normal 6-month-old mice [6]. Control or glycerol-injured mice were twice infused (via the tail vein) with 0.5 million CD_24+_ PKH26 red-stained embryonic kidney cells (or cell supernatant as a control) three and four days after glycerol injection. Kidneys were examined by immunofluorescent microscopy after an additional 3 days. In uninjured mice, proximal tubules showed normal staining for* Lotus tetragonolobus* agglutinin (LTA) and there was no uptake of exogenous CD_24+_ PKH26 red-stained cells into the kidney (Figure 6(a)). In glycerol-injured mice which had no infusion of cells, we noted extensive tubular dilatation, patchy flattening, or necrosis of proximal tubular epithelial cells (Figure 6(b)). No nonspecific uptake of dye was seen in glycerol-injured mice infused with the supernatant from PKH26-stained CD_24+_ cells (Figure 6(c)). However, there was widespread integration of the exogenous embryonic CD_24+_ PKH26 red-stained cells into renal tubules of glycerol-injured mice (Figure 6(d)). At high power, red-stained exogenous CD_24+_ cells were integrated into the proximal tubular wall and exhibited a polarized epithelial phenotype (Figures 6(e) and 6(f)). LTA expression is seen both in the exogenous PKH26 red-stained CD_24+_ cells and in the adjacent endogenous proximal tubular cells (Figure 6(h)).

### 2.4. CD_24+_ Cells from Embryonic *β*-Catenin/TCF Mice Activate the Canonical WNT/*β*-Catenin Signaling Pathway When Infused into Glycerol-Injured Mice

**Figure 7:**
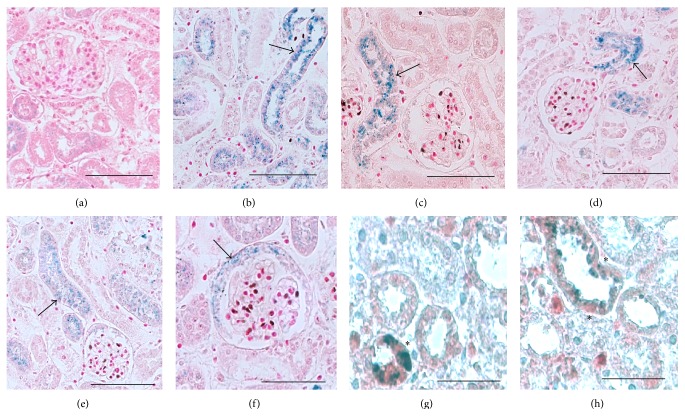
*β*-catenin/TCF pathway reporter activity in exogenous CD_24+_ cells during integration into the glycerol-damaged adult renal tubule. (a–h) CD_24+_ cells (0.5 million) were isolated by FACS from embryonic E15 mice bearing the *β*-catenin/TCF reporter and infused into CD1 wild-type adult mice 3 days after i.m. administration of glycerol 8 *μ*L/g or PBS (control). Kidney sections were stained with X-Gal to identify *β*-catenin/TCF reporter activity (blue) after an additional 3 days. (a) Minimal *β*-catenin/TCF reporter activity is seen after infusion of CD_24+_ cells into control mice. (b-c) Strong *β*-catenin/TCF reporter activity (arrow) is seen in exogenous cells integrated into glycerol-damaged renal tubules. (d-e) *β*-catenin/TCF reporter activity (arrow) is seen in exogenous cells integrated into the S1 segment of glycerol-damaged proximal tubules. (f) *β*-catenin/TCF reporter activity (arrow) is seen in exogenous cells integrated into the urinary pole of Bowman's capsule. (g-h) Exogenous CD_24+_ cells integrated into glycerol-damaged renal tubules exhibit both *β*-catenin/TCF reporter activity and strong staining for the marker of cell proliferation, PCNA (red) (asterisk). Scale bars: 50 *μ*m.

To determine whether exogenous CD_24+_ cells activate the canonical WNT signaling pathway during integration into damaged tubules, we isolated CD_24+_ cells from E15 embryonic *β*-catenin/TCF reporter mice and infused them into adult wild-type mice with glycerol-induced proximal tubular injury. No exogenous reporter signal is seen in kidney of uninjured mice (Figure 7(a)), but robust canonical WNT signaling activity is evident in exogenous cells integrated into many (but not all) tubules of the injured kidney (Figures 7(b)–7(f)). In some sections, the signaling is clearly evident in the S1 segment of proximal tubules at the junctions with renal glomeruli (Figures 7(b)–7(d)). Occasionally, the WNT signaling activity is seen in exogenous cells lining the urinary pole of Bowman's capsule (Figure 7(f)). Exogenous CD_24+_ cells expressing the *β*-catenin/TCF reporter also showed strong staining for the cell proliferation marker, PCNA (Figures 7(g) and 7(h)).

### 2.5. WNT4 Is Activated in Exogenous CD_24+_ Cells during Renal Regeneration

During nephrogenesis, the canonical WNT signaling pathway is initially activated in renal progenitor cells by WNT9b released from the UB; WNT signaling is then sustained in RPC by endogenous WNT4 expression as the cells undergo the mesenchyme-to-epithelial transition and form the S-shaped body [7, 8]. We reasoned that the *β*-catenin/TCF-lacZ reporter activity seen after acute glycerol-induced tubular injury might be driven by reexpression of an autocrine or paracrine WNT signal. As seen in Table 1, E15 embryonic mouse kidney expresses WNTs 4, 7b, 9b, and 11. Of these,* Wnt4* mRNA is detected in CD_24+_ cells and in glycerol-injured adult kidney, but not in uninjured kidney. Wnt9b was not expressed in CD_24+_ cells but was detectable by RT/PCR both in control and in glycerol-injured adult mice.* Wnt11* mRNA was noted in the pool of CD_24+_ cells isolated from embryonic kidney but not in control or glycerol-injured adult kidney. Wnt7 transcripts were detected in all samples.

### 2.6. Activation of the Canonical WNT/*β*-Catenin Pathway Is Required for Integration of CD_24+_ Cells into Damaged Proximal Tubules

**Figure 8:**
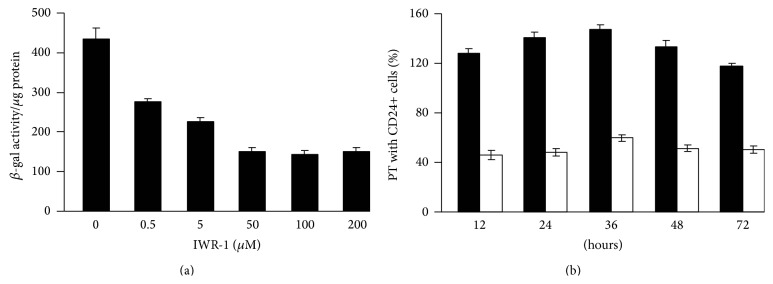
IWR-1 inhibits WNT3a-stimulated *β*-catenin/TCF signaling activity in embryonic CD_24+_ cells from E15 mouse kidney. CD_24+_ cells from mice bearing the *β*-catenin/TCF reporter transgene were isolated by FACS from E15 mouse kidneys and cultured for 12 hours. IWR-1 was then added and CD_24+_ cells were exposed to L-cells expressing WNT3a for 12 hours to assess *β*-catenin/TCF signaling activity. (a) *β*-galactosidase activity per *μ*g cell protein was maximally inhibited to 34% of control by IWR-1 concentrations 100 *μ*M (24 hours). (b) CD_24+_ cells were exposed to control medium (black bars) or IWR-1 (100 *μ*M) (white bars) for 12 hours and then transferred to control medium for various periods of time to track persistence of WNT3a-stimulated *β*-galactosidase activity; significant inhibition was evident after 12 hours (36% of control) and 72 hours (43% of control) of washout in control medium.

To inhibit the WNT/*β*-catenin pathway, we isolated CD_24+_ cells from E15 *β*-catenin/TCF reporter mice and exposed them to IWR-1, an inhibitor of tankyrase proteins that destabilize the derivative axin complex [9]. Previously, investigators have demonstrated that IWR-1 (100 *μ*M) blocks canonical WNT signaling in E11.5 embryonic kidney explants [10]. After 24-hour exposure to 100 *μ*M IWR-1, WNT3a-stimulated *β*-catenin/TCF reporter activity was reduced to 33% of control (Figure 8(a)). To confirm that IWR-1 inhibition persisted during the period during which exogenous cells are integrated into damaged tubules, we exposed CD_24+_ cells bearing the *β*-catenin/TCF reporter to 100 *μ*M IWR-1 for 12 hours and then transferred the cells to control culture medium for various periods of time. After 72 hours, WNT3a-stimulated *β*-catenin/TCF reporter activity was still inhibited to 43% of control (Figure 8(b)).

**Figure 9:**
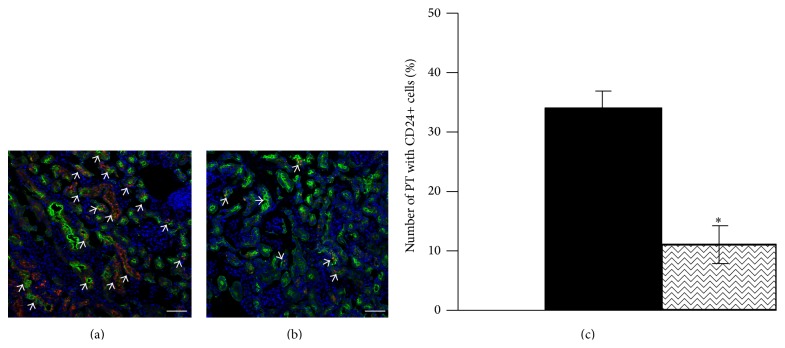
IWR-1 pretreatment of exogenous CD_24+_ cells reduces integration into glycerol-damaged renal proximal tubules. CD_24+_ cells were isolated by FACS from embryonic E15 mouse kidney and cultured for 12 hours in the presence or absence of IWR-1 (100 *μ*M). 0.5 million CD_24+_ cells were then labeled with PKH26 red dye and infused into mice three days after glycerol-induced proximal tubular injury. (a) Control CD_24+_ cells (white arrows) are widely integrated into damaged proximal tubules stained with LTA (green); (b) proximal tubular integration (white arrows) of CD_24+_ cells after IWR-1 pretreatment is strikingly reduced (a-b, scale bars: 50 *μ*m). (c) The number of LTA-stained proximal tubules (PT) showing integration of PKH26 red CD_24+_ cells was estimated in 310–340 tubules from 10 different kidney sections. Pretreatment of exogenous CD_24+_ cells with IWR-1 (100 *μ*M) reduced the percentage of tubules showing CD_24+_ cells integration from 34% (control) to 11% (IWR-1). *P* < 0.001.

To examine the effect of IWR-1 pretreatment on exogenous cell integration into damaged adult tubules, we isolated CD_24+_ cells from wild-type embryonic E15 mouse kidney, cultured them for 12 hours in the presence or absence of IWR-1 (100 *μ*M), and then infused 0.5 million PKH26 red-labeled cells into mice three days after glycerol-induced proximal tubular injury. Whereas control CD_24+_ cells are widely integrated into damaged proximal tubule segments, integration of IWR-1 pretreated CD_24+_ cells was strikingly reduced (Figure 9(b)) compared to untreated cells (Figure 9(a)). The percentage of proximal tubules exhibiting exogenous CD_24+_ cell integration fell from 34% (untreated cells) to 11% (IWR-1 pretreated cells) (Figure 9(c)).

## 3. Discussion

There are over 300 human clinical trials of adult mesenchymal stem cells registered at the NIH clinical trial registry (http://clinicaltrials.gov/). These studies are predicated on the ability of infused adult bone marrow stem cells to home to sites of acute tissue injury and exert a number of salutary effects on the tissue injury/repair process. However, although kidney is derived from stem cells within the embryonic mesenchyme, adult mesenchymal stem cells lack the capacity for integration into the intrinsic epithelial structures of adult organs. Infusion of bone marrow MSC hastens the recovery from experimental kidney injury, but the exogenous cells take up an interstitial position adjacent to renal tubules and are rarely incorporated into the tubular wall [11, 12]. Similarly, infusion of bone marrow hematopoietic stem cells into CTNS knockout mice with nephropathic cystinosis can reverse pathologic cystine accumulation and ameliorate progressive renal dysfunction [13]. Again, however, these cells were noted to take up a peritubular position [13] and the effect on adjacent mutant cells was recently attributed to paracrine transfer of wild-type cystinosin via microvesicle shedding [14].

In contrast to the above, Lazzeri et al. infused human kidney embryonic CD_24+_/CD_133+_ cells into glycerol-injured adult SCID mice and demonstrated accelerated renal recovery associated with widespread integration of the exogenous cells into damaged renal tubules [6]. Thus, while nephron progenitor cells in the metanephric mesenchyme may have lost some of the plasticity exhibited by embryonic stem cells, they appear to have acquired special characteristics which facilitate their integration into nephrons during primary nephrogenesis. Although stem cells from bone marrow also arise from embryonic mesenchyme, they have not acquired (or have lost) these characteristics. In our studies, embryonic CD_24+_ cells show responsiveness to WNT3a* in vitro* and when infused into mice with glycerol-induced injury, they exhibit robust activation of the pathway during integration into the tubular wall. Importantly, IWR-1 inhibition of the *β*-catenin/TCF pathway sharply reduces integration of exogenous NPC into the damaged tubules. Taken together, these observations suggest that embryonic NPC have been primed to activate the *β*-catenin/TCF pathway in response to an inductive WNT signal and that this capacity is crucial for their ability to regenerate damaged adult renal tubules.

We used FACS for the CD24 surface marker to capture a population of cells from embryonic kidney with the capacity to integrate into nephrons. In E10.5 embryonic mice (prior to nephrogenesis),* Cd24* mRNA is strongly and specifically expressed in the metanephric mesenchyme [15]. As reported by others at E15, we noted CD24 protein expression in cells of the cap mesenchyme surrounding each ureteric bud tip, in cells undergoing the mesenchyme-to-epithelium transition in the S-shaped body, and in a few cells of the branching ureteric bud [16, 17]. Thus, our E15 CD_24+_ cells comprise a heterogeneous mix of uninduced RPC (expressing* Osr1*,* Wt1*, and* Six2*) and cells in transition to the epithelial phenotype of proximal tubules (*Wnt4* and* Pax8*) and collecting ducts (*Wnt7b*). Few E15 CD_24+_ cells from embryos with the *β*-catenin/TCF reporter exhibited basal activity of the canonical WNT signaling pathway. However, about 40% displayed *β*-catenin/TCF reporter activity in response to WNT3a or cocultured UB cells. Several days after infusion into adult mice with glycerol-induced renal injury, a substantial number of CD_24+_ cells are seen within the tubular wall, displaying a polarized epithelial phenotype and luminal expression of LTA, a marker of proximal tubular cell differentiation. Thus, the pool of CD_24+_ cells isolated from E15 kidney contains a subset of functional NPC that respond to canonical WNT signals, express genes involved in tubular differentiation, integrate into to the nephron wall, and exhibit properties of the mature tubular cell* in vivo*. In humans, the progenitor cell pool can be further refined by coselection for the CD133 antigen, noted in the cap mesenchyme and S-shaped body [6], but an antibody for the murine homolog of CD133 has not been developed. In mice, it may be possible to distinguish compartments of uninduced self-renewing RPC (Six2+/Cited1+) from WNT-inducible NPC (Six2+) in the cap mesenchyme [18]. However, our studies do not attempt to characterize the specific subset of NPC that integrate into damaged tubules but, rather, focus on the *β*-catenin-dependent mechanism involved in integration of these cells.

During primary nephrogenesis, it is thought that NPC in the cap mesenchyme begin to differentiate in response to WNT9b released from ureteric bud tips [3]. However, during progression to the S-shaped body stage, autonomous expression of WNT4 is required to sustain canonical pathway activity and complete nephrogenesis [7]. WNT7b is expressed in the ureteric bud trunk and WNT11 at its tip. However, mammalian nephrogenesis comes to an end in the perinatal period and embryonic WNT expression is downregulated in adult kidney. Thus, it is unclear how the canonical WNT/*β*-catenin signaling pathway is activated in exogenous or endogenous CD_24+_ cells that can repopulate the damaged renal tubule. Of the four potential canonical WNT ligands expressed in embryonic kidney (WNTs 4, 7b, 9b, and 11), transcripts for all except* Wnt9b* were detectable by RT/PCR in embryonic CD_24+_ cells. Interestingly,* Wnt4* mRNA is induced by glycerol injury in adult kidney. Although our studies do not implicate any one particular WNT, it is clear that both embryonic CD_24+_ cells and damaged adult kidney express several ligands that could account for pathway activity.

Our studies demonstrate *β*-catenin/TCF reporter activity not only in the exogenous embryonic CD_24+_ cells which are taken up by damaged renal tubules, but also by endogenous tubular cells that express CD24 after glycerol injury. The origin of these endogenous cells is unclear. Humphreys has argued that recovery from acute tubular necrosis cannot be explained by new tubular cells arising from a single stem cell focus in each nephron [19]. However, Angelotti et al. has identified subsets of apoptosis-resistant CD_24+_/CD_133+_/CD_106+_ cells retained in putative stem cell niches at the urine pole of Bowman's capsule and related CD_24+_/CD_133+_/CD_106-_ cells scattered along the proximal and distal nephron [20]. When isolated from adult kidney by FACS, these cells express* OSR1* [21], exhibit self-renewal* in vitro*, and can be induced to express a variety of markers of the proximal tubule, loop of Henle, and distal convoluted tubule [4]. These observations favor the view that adult mammalian nephrons retain a population of quiescent NPC that participate in regeneration of the damaged nephron. Following glycerol-induced tubular injury in adult *β*-catenin/TCF reporter mice, we noted striking endogenous *β*-catenin/TCF signaling activity both in the crescent of cells lining the urinary pole of Bowman's capsule and in the proximal tubule. Although this mirrors the two subsets of cells described by Angelotti et al. [20], formal fate-mapping studies are needed to ascertain whether discrete subsets of adult NPC repopulate the damaged adult tubule or whether all tubular cells can dedifferentiate, express CD24, and reactivate the WNT4/*β*-catenin/TCF pathway when injured.

In summary, our observations indicate that a crucial distinction between the committed nephron progenitor cell in metanephric mesenchyme and the mesenchymal stem cell from adult bone marrow is the capacity to activate the *β*-catenin/TCF pathway in response to canonical WNT signals. We suggest that this property may be essential for any cell-based regenerative therapy of the damaged mammalian kidney.

## 4. Materials and Methods

### 4.1. Isolation and Culture of Embryonic E15 CD_24+_ Cells

To obtain embryonic CD_24+_ cells, kidneys were resected from embryonic day E15 wild-type or transgenic mice bearing a *β*-catenin/TCF-responsive beta-galactosidase reporter transgene (*β*-catenin/TCF-lacZ) [2]. Embryonic mouse kidneys were minced and digested with 1 mg/mL collagenase type B (Roche), 2.5 mg/mL Dispase II (Roche), and 30 *μ*g/mL DNase I in F12/DMEM culture medium with 10% FBS at 37°C for 45 minutes under 5% CO_2_. The cell suspension was filtered through a 35 *μ*m cell strainer in a 15 mL Falcon tube, washed three times with cold 1X PBS containing 2% FBS, and pelleted at 1000 rpm/min for 5 min (4°C). The cells were then placed in monolayer culture (F12/DMEM + 10% FBS at 37°C under CO_2_) for 24–48 hours. The adherent E15 monolayer culture was then detached with trypsin, and the single-cell suspension was incubated with CD24 antibody conjugated to Alexa Fluor 647 (Biolegend) in the dark. CD_24+_ cells were isolated by fluorescence-activated cell sorting (FACS) with forward and side scatter width/height gating to ensure isolation of singlet cells. Cell sorting was performed on a MoFlo cell sorter (DakoCytomation, Carpinteria, CA). In some experiments, the CD_24+_ cells were stained with PKH26 red (Sigma-Aldrich) according to manufacturer's instructions and washed three times in chilled PBS prior to use. In other experiments, they were exposed to various concentrations of the tankyrase inhibitor IWR-1 (Enzo Life Science).

### 4.2. *β*-Galactosidase Activity of CD_24+_ Cells* In Vitro*

*β*-galactosidase activity generated by the TCF/*β*-gal reporter transgene was determined using the Tropix Galacto-Star system chemiluminescent reporter gene assay system (Applied Biosystems). The signal was measured in a GLOMAX 96 microplate luminometer (Promega, San Luis Obispo, CA, USA).

### 4.3. Activation of Canonical WNT Signaling Pathway in Cultured CD_24+_ Cells

To activate the canonical WNT signaling pathway* in vitro*, monolayer cultures of E15 CD_24+_ cells bearing the *β*-catenin/TCF reporter were exposed for 24 hours to coculture inserts (0.4 *μ*m pore size) containing (1) mouse fibroblast L-cells (ATCC); (2) L-cells expressing WNT3a (ATCC); (3)* Hoxb7*-GFP(+) ureteric bud cells isolated by FACS from E15* Hoxb7*-GFP C3H mice. These mice express the* Hoxb7*-GFP transgene exclusively in the ureteric bud lineage in embryonic kidney [22, 23].

### 4.4. Analysis of *β*-Galactosidase Activity in Transgenic Mice

Whole kidneys or monolayer cultures of E15 CD_24+_ cells from mice bearing the *β*-catenin/TCF-LacZ reporter transgene were fixed and stained as previously described [2]. Kidneys were washed in PBS and visualized directly or embedded in paraffin for sectioning, counterstained with hematoxylin and eosin, and visualized by light microscopy.

### 4.5. Glycerol-Induced Acute Renal Tubular Injury in Mice

Proximal renal tubular injury was induced with intramuscular injection of 50% glycerol (8 *μ*L/g body weight) (Sigma-Aldrich) into the inferior hind limbs of normal 6-month-old CD1 mice (Charles River Lab, USA) under anesthesia as described by Lazzeri et al. [6]. Control mice were injected with the same volume of phosphate buffered saline. Control or glycerol-treated mice were twice infused via the tail vein with 0.5 million embryonic CD_24+_ cells three and four days after glycerol injection: Group 1 (*n* = 8) received two intravenous infusions of embryonic CD_24+_ cells labeled with PKH26 red fluorescent dye; as a control in a parallel experiment, mice were injected with supernatant from the third saline wash of PKH26-stained cells; Group 2 (*n* = 8) received two intravenous infusions of CD_24+_ cells obtained from E15 *β*-catenin/TCF transgenic embryonic mice [2].

To study the effect of acute renal injury on endogenous canonical WNT signaling pathway activity, we induced acute proximal tubule injury with glycerol in six-month-old CD1 mice (*n* = 8) bearing the *β*-catenin/TCF reporter transgene. Three days after the first infusion (day 6), mice were killed and kidneys were harvested for analysis.

All animal procedures followed the guidelines established by the Canadian Council of Animal Care and were approved by the Animal Care Committee from the Research Institute from the McGill University Health Center.

### 4.6. Reverse Transcriptase PCR

Total RNA was isolated from cells using Qiagen RNeasy Mini-Plus Kit with gDNA eliminator column (Qiagen, Mississauga, ON, Canada). Two-step reverse transcriptase-PCR was performed; first-strand cDNA was primed with random hexamers and TaqMan MultiScribe Reverse Transcriptase according to the manufacturer's instructions (Applied Biosystems, Foster City, CA).

### 4.7. Immunohistochemistry

Paraffin-embedded sections (5 *μ*m) of embryonic or adult kidneys were incubated in 5% H_2_O_2_ to quench endogenous peroxidase activity, followed by 30 min incubation with normal horse serum. Tissue sections were then incubated with rabbit polyclonal antibody against murine PCNA (1 : 100) (Santa Cruz, CA) at 4°C overnight and then incubated with a universal biotinylated secondary antibody (Vector Laboratory, Burlingame, CA). Staining was developed using ACE (Vector Laboratory).

### 4.8. Immunofluorescent Staining

Staining was performed on 14 *μ*m frozen sections of embryonic mouse kidney. Briefly, sections were rinsed in PBS for 10 min and fixed in ice-cold acetone for 10 min. Sections were blocked with horse serum for 1 h at room temperature and then incubated with anti-CD24 PE conjugated antibody (1 : 200), counterstained with LTA and DAPI, and mounted with aqueous gel mount (Sigma). Confocal microscopy was performed with a Zeiss LSM780 Laser Scanning Confocal Microscopy (Carl Zeiss, Jena, Germany).

### 4.9. Statistical Analysis

The results are expressed as the mean ± s.d. Statistical significance between experimental groups was assessed by Student's unpaired *t*-test.

## Figures and Tables

**Table 1:** *Wnt* mRNA expression in mouse embryonic and adult kidney.

	Wnt4	Wnt7b	Wnt9b	Wnt11
E15 mouse kidney	+	+	+	+
CD_24+_ cells (E15)	+	+	No	+
Adult kidney (no glycerol)	No	+	+	No
Adult kidney (with glycerol)	+	+	+	No

Transcripts for each *Wnt* were identified by RT/PCR in E15 mouse kidney, CD24 cells isolated by FACS from E15 mouse kidney, normal adult mouse kidney, and adult mouse kidney 3 days after induction of glycerol-induced proximal tubular injury.
